# Insights into the Donkey Hindgut Microbiome Using Metagenome-Assembled Genomes

**DOI:** 10.3390/ani14243625

**Published:** 2024-12-16

**Authors:** Xiyan Kou, Yihong Liu, Fokun Xiang, Xinyue Zhang, Muhammad Zahoor Khan, Boxian Wu, Hua Wang, Yanlin Gong, Changfa Wang, Qingshan Ma, Yan Li

**Affiliations:** 1 School of Agricultural Science and Engineering, Liaocheng University, Liaocheng 252000, China; 2 Shandong Dong’e Black Donkey Husbandry Technology Co., Ltd., Liaocheng 252000, China

**Keywords:** metagenome-assembled genomes, CAZymes, donkey, gut microbiome, metagenomics

## Abstract

The gut microbiota plays an important role in the digestion, absorption, and metabolism of nutrients, as well as in the immunity, health, and behavior of donkeys. While reference genomes and gut microbial gene catalogs have been helpful in understanding the composition of the donkey, there is still a significant gap in sequencing and understanding the functional aspects of donkey gut microbial genomes. In this study, we analyzed metagenomic sequencing data from 26 donkeys’ gut samples and successfully assembled 844 microbial metagenome-assembled genomes (MAGs). Surprisingly, 678 (80.33%) of these MAGs appear to belong to previously unidentified species. Our analysis further revealed a total of 292,980 predicted carbohydrate-active enzymes (CAZymes) and 257,893 polysaccharide utilization loci (PULs). Interestingly, these enzymes and loci displayed relatively low similarity matches in public databases. We found that the higher abundances of 36 MAGs in the cecum (such as *Prevotella*, *Desulfovibrio*, *Alistipes*, and *Treponema_D*) and 9 MAGs in the dorsal colon (such as *Limimorpha*, *Saccharofermentans*, and *Lactobacillus*) were associated with a diverse array of carbohydrate-degrading pathways. Network analysis identified *Prevotella* and *Dysosmobacter* as connectors, while *Saccharofermentans* and *Akkermansia* were shown as provincial hubs. This suggests their crucial roles in complex carbohydrate degradation and hindgut metabolism in donkeys. These findings underscore the complexity of hindgut metabolism in donkeys and expand our understanding of their gut microbiome. Overall, this study provides a comprehensive catalog of donkey gut microbial genes, revealing novel carbohydrate-degrading enzymes and offering new insights for future research on the donkey gut microbiome.

## Simple Summary

Numerous microorganisms found in the gastrointestinal tracts of donkeys are challenging to cultivate due to their unique and often unknown growth requirements. This study provides a comprehensive catalog of donkey gut microbial genes and expands our understanding of the donkey gut microbiome. For the first time, metagenome-assembled genomes from the donkey hindgut have been characterized. Our dataset serves as a valuable resource for the discovery of novel carbohydrate-degrading enzymes and for further research on the donkey gut microbiome.

## 1. Introduction

With the rapid advances in microbiology and biotechnology, the role of the gut microbiome in animal health has received widespread attention [1,2]. Especially in the donkey, a species with unique physiological characteristics and significant economic value, it is important to explore its gut microbiome. The donkey is a rare species endemic to China, and products derived from it, such as Colla Corii Asini, donkey meat, and donkey milk, are in high demand in the market [3]. However, under the trend of increasing market demand, the healthy and efficient breeding of donkeys is becoming more and more critical. Gut microorganisms, as a large and complex micro-ecosystem in the animal body, build a close symbiotic relationship with the host [4]. These microorganisms not only participate in the digestion and metabolism of nutrients, but also influence the development of the digestive tract and the immunomodulation of the host [5,6]. Donkey gut microorganisms are diverse, and their distribution is closely related to their functional areas. Significant differences in microbial composition and function between the cecum and the colon highlight the critical role of gut microbes at all stages of digestion [7]. An in-depth understanding of the structure and function of the donkey gut microbiome could not only improve the gut health of donkeys, but also enhance their performance; however, there are still few reports on the donkey gut microbiota.

The donkey hindgut is highly developed and consists of the cecum, colon, and rectum [8]. The cecum and colon constitute over 60% of the gastrointestinal (GI) tract [9] and serve as the main reservoir for a diverse microbial community, including bacteria, anaerobic fungi, methanogenic archaea, and ciliate protozoa [10]. This intestinal microbiota and its host have co-evolved into a complex and mutually adaptive micro-ecological system [11], which plays a critical role in nutritional metabolism, the immune system [12], feed efficiency, and the health of donkeys [13]. Previous studies have utilized 16S rRNA gene high-throughput sequencing technology to investigate the microbial diversity and function of the donkey intestine across different sections [14], ecological sites (liquid phase and adherent fraction) [15], ages [16], as well as before and after weaning [13], and at various stages of gestation [17]. Despite these efforts, our understanding of the intestinal microbial community in donkeys still lacks comprehensive insight.

Many of the microorganisms found in the gastrointestinal tract of donkeys are challenging to cultivate due to their distinct and often unknown requirements for growth [18]. Recent advancements in metagenomics, particularly metagenome-assembled genomes (MAGs) reconstructed from metagenomic sequences, have significantly enhanced our understanding of microbial genomes and novel enzymes, such as those classified under carbohydrate-active enzymes (CAZymes). These enzymes have the function of degrading synthetic carbohydrates and their derivatives, in various hosts including humans, cattle, sheep, and horses [19,20,21,22]. Despite these advancements, reports on the donkey gut microbiota remain infrequent.

Previously, we sought to evaluate and compare the microbial community and its function in the cecum and colon of donkeys using metagenome sequencing [18]. In this current study, we expanded our sample size by including an additional 16 donkey hindgut samples. We then assembled the metagenomic sequences and grouped them into MAGs based on their completeness (≥50%) and contamination (≤10%). Our main objective was to characterize the functional potential of these MAGs, specifically focusing on CAZymes, polysaccharide utilization loci (PULs), and KEGG analyses. We also aimed to associate these activities with specific taxa. Additionally, we investigated the differential MAGs in the hindgut and examined the co-occurrence and interaction networks of MAGs in the cecum.

## 2. Materials and Methods

### 2.1. Animal Selection, Husbandry, and Sample Collection

In this study, 26 samples were collected from healthy donkeys aged between 2.5 and 3 years; details are provided in Supplementary Table S1. All donkeys were raised under uniform farming conditions at a Dezhou donkey-breeding farm authorized by Shandong Province (Dezhou city, Shandong, China). The donkeys were fed ad libitum with a corn straw diet supplemented by a commercial concentrate (Hekangyuan Group Co., Ltd., Jinan, China) and were fed twice daily at 07:00 and 19:00. They had continuous access to clean water. Additionally, none of the donkeys had any previous history of gastrointestinal disorders, and they had not been administered antibiotics for at least three months prior to sampling. The animal care protocol used in this study adhered to commercial management practices and was approved by the Animal Welfare Committee of Liaocheng University (Permit No. DFG21010103-1).

Donkeys for non-research purposes were euthanized at a local abattoir. After a 12 h fasting period, the animals were stunned using electronarcosis at 220 V for 20 s, then slaughtered via exsanguination using standard humane method [23]. Based on the anatomical differences along the donkey hindgut, we collected 26 samples of cecal contents from 26 donkeys, as well as additional samples from the ventral colon and dorsal colon of 4 of these animals. After externalization of the gastrointestinal tract, the cecum, ventral colon, and dorsal colon were bundled to avoid mixing between adjacent segments. Immediately, the hindgut contents of one segment were collected by press-filtering with four layers of clean gauze and mixed thoroughly to obtain representative samples of the cecum, ventral colon, and dorsal colon. All samples were transferred to separate, sterilized 2 mL tubes and then frozen immediately in liquid nitrogen. Subsequently, all frozen samples were transported on dry ice to the laboratory and stored at −80 °C for further analysis.

### 2.2. Genomic DNA and Genome Sequencing

Total genomic DNA was extracted from 100 mg of frozen contents from the hindgut content of donkeys using an E.Z.N.A.^®^ Soil DNA Kit (Omega Bio-Tek, Norcross, GA, USA) according to the manufacturer’s instructions. DNA yield and quality were determined with a NanoDrop2000 (Thermo Scientific, Wilmington, NC, USA). DNA fragments with an average size of approximately 400 bp were sequenced on an Illumina NovaSeq/HiSeq XTen instrument (Illumina Inc., San Diego, CA, USA) at Majorbio Bio-Pharm Technology Co., Ltd. (Shanghai, China) using NovaSeq Reagent Kits/HiSeq X Reagent Kits according to the manufacturer’s instructions (www.illumina.com).

### 2.3. Metagenomic Assembly and Binning

The data were analyzed using the Majorbio Cloud Platform (www.majorbio.com) as detailed by Ren et al. [24]. To generate clean reads from metagenome sequencing, raw reads were processed with fastp (version 0.20.0) [25]. This involved removing adaptor sequences, trimming and eliminating low-quality reads (reads with N bases, shorter than 50 bp or with an average quality score < 20). Reads were aligned to the Dezhou donkey genome (Genome assembly ASM1607732v2) by BWA (version 0.7.9a) and any hit associated with the reads and their mated reads were removed. Then, these high-quality reads were assembled into contigs using MEGAHIT (version 1.1.2) [26], which utilizes succinct de Bruijn graphs (SdBGs). Contigs with a length of 1000 bp or longer were selected as the final assembly result. These contigs were further analyzed for binning to retrieve metagenome-assembled genomes (MAGs). For calculating the relative abundance of each MAG, CoverM (version 0.6.1) was used with the option -m relative_abundance covered_fraction.

The binning was performed using MetaBAT (version 2.12.1) [27], CONCOCT (version 0.5.0) [28], and MaxBin2 (version 2.25) [29]. The completeness, contamination, and strain heterogeneity of the MAGs were estimated by CheckM (v. 1.1.2) [30], and the MAGs with more than 50% completeness and less than 10% contamination were kept for downstream analysis. dRep (version 2.6.2) [31] was used to eliminate low-quality and redundant MAGs with the clustering at the threshold of 99% ANI with at least 30% (i.e., parameters set as -comp 50-con 10-sa 0.99-nc 0.30) overlap between genomes.

### 2.4. Annotation and Functional Analyses of MAGs

GTDB-Tk (version 2.3.2) [32] was performed to assign the taxonomy of the MAGs. ORFs were predicted from the assembled MAG using MetaGeneMark (version 3.38). Protein sequences were aligned to the eggNOG database (version 5.0.2) using the eggnog-mapper (version 2.1.7) [33]. The alignment software utilized by eggnog-mapper was Diamond (version 2.0.15), enabling the acquisition of KEGG and GO annotations. Protein sequences were aligned to the CAZymes (carbohydrate-active enzymes) in the dbcan2 database (https://bcb.unl.edu/dbCAN_PUL/) (accessed on 20 March·2024) using Diamond, with an e-value of ≤1 × 10^−3^ and the highest similarity [34]. Diamond was used to align protein sequences with the PULs from the DBCAN-PUL database (https://bcb.unl.edu/dbCAN_PUL/) (accessed on 20 March 2024) [35], the best similarity alignments, and findings with an e-value less than 1e-3 were kept. Phylogenetic trees were constructed using the “infer” in GTDB-Tk for two independent sets of MAGs, and the R package ggtree was used to visualize the results [36]. In addition, we utilized the clusterProfiler R program to examine KEGG analysis among various MAGs [37]. To simplify the functional annotations, we derived the metabolic capacity index (MCI), aggregate genome-inferred functional traits (GIFTs), and community-weighted average GIFTs of genomes using the R package distillR (version 0.3.0). Moreover, we utilized the R package pheatmap (version 1.0.12) to display the proportion of GIFTs in samples and genomes.

### 2.5. Analyses of Interaction Networks of MAGs

MAGs exhibiting an average relative abundance exceeding 0.005% in cecum samples were selected for analysis. Spearman correlations were computed for MAGs within each defined group utilizing using R. Results were subjected to filtration based on correlations > 0.85 and Bonferroni-corrected *p*-values < 0.05. Moreover, to visualize the network, the graph package in R was utilized. Furthermore, to identify core MAGs, network characteristics, z-score, and c-score were utilized. The z-score is used to measure the degree of difference between the number of connections observed in the network and the number of connections expected in a randomized network, and the c-score is used to measure the uniformity of the node’s connections across modules.

### 2.6. Statistical Analysis

All statistical analyses were conducted using R (version 4.0.1). α diversity (richness index, Shannon index, and Simpson index) was calculated using Usearch10 (https://www.drive5.com/usearch/manual/citation.html) (accessed on 25 March 2024) [38]. The Tukey test was used for the statistical analysis. Beta diversity was assessed using Principal Coordinate Analysis (PCoA) based on Bray–Curtis dissimilarity matrices. The PCoA plots displayed the differences in beta diversity among the cecum (C), ventral colon (VC), and dorsal colon (DC) from four donkeys. Constrained Principal Coordinate Analysis (cPCoA) based on Bray–Curtis dissimilarity was used to visualize differences in community structure between samples, and the PERMANOVA test was applied to quantify statistically significant differences between groups in the PCoA plot. The differences in the abundance of MAGs between two groups were analyzed using Mann–Whitney test and paired *t*-test. Means were considered significantly different when *p* < 0.05. Diagrams were generated using the R package amplicon (version 1.19.0), and box and scatter plots were created using the ggplot2 library.

### 2.7. Data Availability

The unassembled sequences as well as the MAGs from the present study were submitted to the NCBI SRA under accession number PRJNA860652 and PRJNA1103961. Scripts are available in GitHub repository (https://github.com/sunichmd/DonkeyGutMetageome_MAG_Analysis) (accessed on 29 March 2024).

## 3. Results

### 3.1. Metagenome-Assembled Genomes

**Figure 1:**
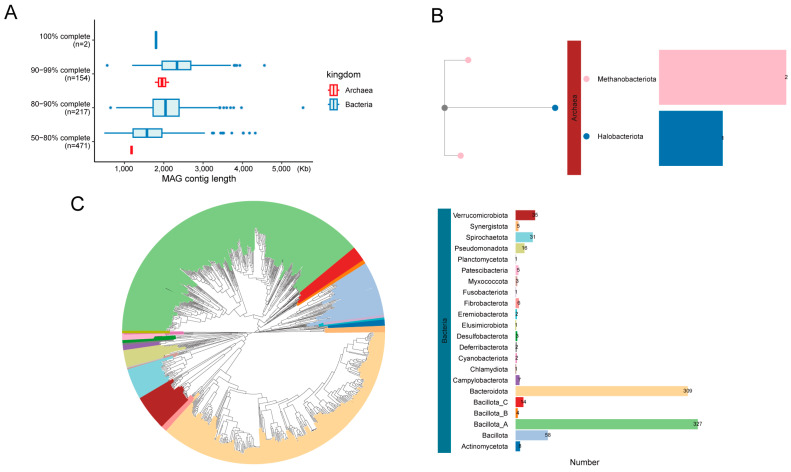
Basic information of assembled MAGs. Distribution of genomic integrity and quality classification of MAGs (**A**). Archaea (**B**) and bacteria (**C**) phylogenetic tree of 844 MAGs from the donkey hindgut.

We obtained 408 Gb of Illumina sequencing data from 34 Dezhou donkey hindgut samples. A metagenomic assembly was performed on each individual sample, resulting in a total of 1046 MAGs (completeness ≥ 50% and contamination ≤ 10%). These 1046 MAGs were then dereplicated using dRep, with a 99% ANI threshold, resulting in 844 non-redundant MAGs (Figure 1A). These non-redundant MAGs consisted of 841 bacteria and 3 archaea (Supplementary Table S2). The total length of the MAGs ranged from 0.49 to 5.54 Mb, with an average length of 1.91 Mb (SEM ± 0.01). The N50 value varied from 1582 to 147,576 bp. ANI calculations were performed between the MAGs and their closest relative genomes from the Genome Taxonomy Database (GTDB). This analysis revealed that 678 MAGs (ANI < 95%) represented undescribed species, and among these, 309 also contained unmapped sequences (Supplementary Table S2). The phylogenetic analysis of the MAGs revealed that, out of the 844 MAGs, 24 distinct phyla were identified, comprising 22 bacterial phyla and 2 archaeal phyla, both of which were methanogenic archaea (Figure 1B,C). In terms of MAG richness within the genus, *Cryptobacteroides* had the highest number of MAGs (57), followed by *Prevotella* (43), *Eubacterium* (31), and *Treponema* (30) (Supplementary Table S2).

### 3.2. Novel CAZymes of MAGs

**Figure 2:**
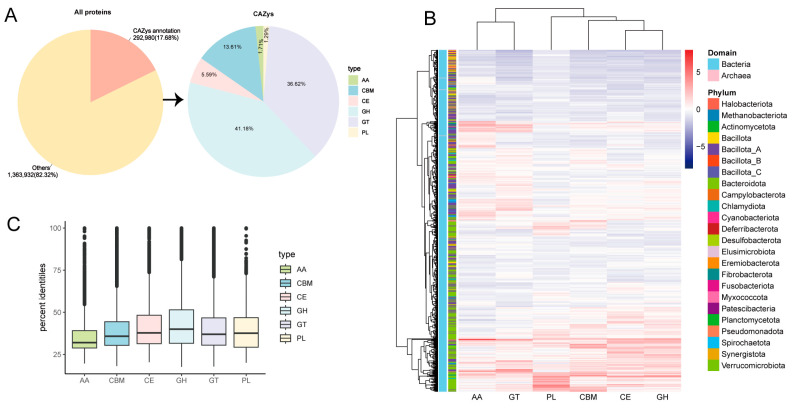
CAZyme annotation of MAGs in the donkey gut. Pie charts of annotation results obtained using dbCAN2 (**A**). Heatmap of the CAZyme distribution (**B**). Sequence similarity between CAZymes in this study and public databases (**C**). Proteins with auxiliary activity (AA), carbohydrate-binding modules (CBMs), carboxyesterases (CEs), glycosyl hydrolases (GHs), glycosyl transferases (GTs), and polysaccharide lyases (PLs).

Among 1,656,912 predicted proteins, 292,980 (17.68%) CAZymes were identified. Based on the analysis, glycosyl hydrolases (GHs) make up 41.18% of the total number of CAZymes, grouped in 6 families, followed by glycosyl transferases (GTs) (36.62%), carbohydrate-binding modules (CBMs), including *Cryptobacteroides* sp018064625, *Cryptobacteroides* sp018064625, and *Faecalibacterium duncaniae* (13.61%), carboxyesterases (CEs) (5.59%), proteins with auxiliary activity, including *Phocaeicola faecalis,* the uncultured species of *Novosphingobium* genus, the uncultured genus KLE1615 of Lachnospiraceae family (AA) (1.71%), and polysaccharide lyases (PLs) (1.29%) (Figure 2A and Supplementary Table S3). The distribution of CAZymes on different annotated MAGs was also analyzed, where AA and GTs were mainly distributed in Bacillota_A, and PL, CBM, CE, and GH distribution was dominated by Bacteroidota (Figure 2B). Further, species belonging to the uncultured genus RUG572 of Kiritimatiellia class (1207), the uncultured family UBA1067 of Kiritimatiellia class (913), *Phocaeicola faecalis* (837), the uncultured species of *Novosphingobium* genus (826), the uncultured genus UBA4334 of Bacteroidaceae family (809), the uncultured genus KLE1615 of Lachnospiraceae family (761), *Escherichia coli* (751), the uncultured species of Prevotella genus (738), the uncultured species of Agathobacter genus (734), the uncultured species of *Cryptobacteroides* genus (669), and *Fibrobacter* sp003149045 (668) possessed the highest proportion of carbohydrate-active enzyme genes (Supplementary Table S3). Protein sequences of MAGs were aligned to the eggNOG database using the eggnog-mapper. Interestingly, high numbers of the degradative enzymes were observed, such as Verrucomicrobiota (MAG49, MAG623, and MAG933) annotated CAZymes, including cellulase (GH5 and GH9), fucosidase (GH29), and pectate lyase (PL1); *Prevotella* (e.g., MAG19, MAG 207, MAG 376, MAG 737, and MAG 756) annotated CAZymes, such as α-amylases (GH13 and GH31), mannosidase (GH38), α-arabinofuranosidase (GH51), α-fucosidase (GH29 and GH18), β-xylanase (GH43), xylan esterase (GH26), 1,4-α-glucan branching enzyme (CBM48), and cellulase (GH5, GH9, and GH26). *Phocaeicola faecalis* possesses certain CAZymes including GH20, GH29, PL8, and PL8_3 (Supplementary Table S4). We further analyze the similarity of the predicted CAZymes against the current CAZyme database. Of the 292,980 CAZyme proteins, only 876 showed highly similar matches with a consistency greater than 95%, indicating that 292,104 are novel CAZyme proteins (average similarity of 41%). Figure 2C suggests that most of the proteins annotated in the donkey hindgut are new CAZymes, which may be highly related to the donkey’s tolerance of rough feeding.

### 3.3. Annotation PULs of MAGs

**Figure 3:**
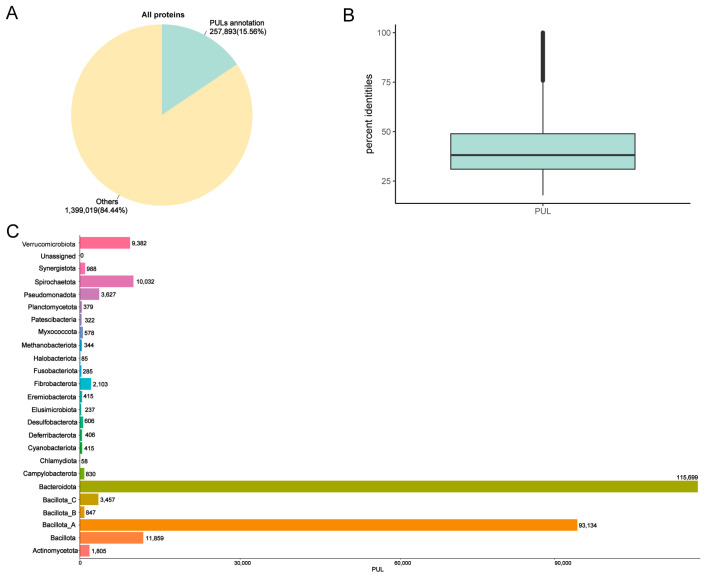
PUL annotations of MAGs in the donkey gut. Annotation results obtained using DBCAN-PUL database (**A**). Sequence similarity between polysaccharide utilization loci (PULs) in this study and public databases (**B**). Distribution of PULs in the phylum (**C**).

CAZymes are often organized in polysaccharide utilization loci (PULs), which consist of clusters of genes responsible for synthesizing and breaking down complex polysaccharides. These loci can be specific to a single substrate or capable of targeting multiple substrates. We identified 257,893 PULs (Figure 3A and Supplementary Table S5). Among these PULs, only 144 showed highly similar matches with >95% consistency, indicating that 257,749 of the identified PULs were unique (average similarity of 41.2%) (Figure 3B). The statistical graph of the number of PULs in phylum–genera revealed that the main PULs were distributed in *Bacteroidota* and *Bacillota_A*, which aligned with the previous CAZyme results. Additionally, the genus *Cryptobacteroides* (21,074) had the highest proportion of PUL gene clusters, followed by *Prevotella* (20,719), UBA4372 (10,187), and *Treponema_D* (9690). At the species level, *Phocaeicola faecalis* had the highest number of PULs (914) (Figure 3C and Supplementary Table S5).

### 3.4. KEGG and MCI Analyses of Hindgut MAGs

**Figure 4:**
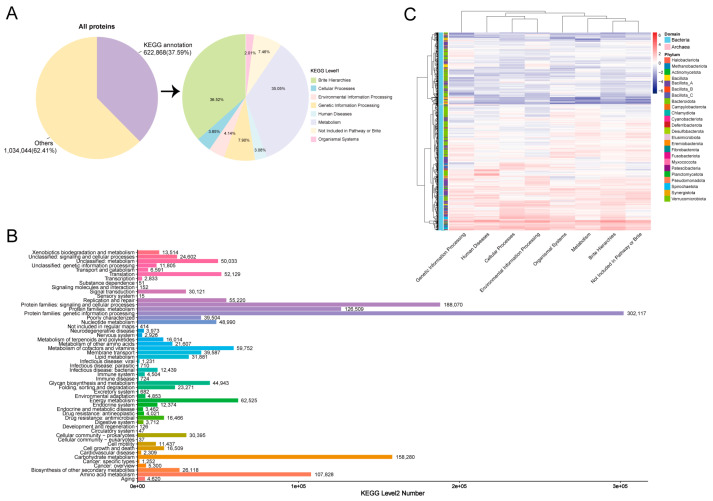
KEGG pathway annotations of MAGs in the donkey gut. Annotation results obtained using KEGG (**A**). KEGG pathway annotations in level 2 (**B**). Distribution of KEGG pathways in the phylum (**C**).

We then conducted KEGG searches to analyze the proteome contents and functions of the MAGs in the donkey hindgut. A total of 1,656,912 proteins were predicted, of which 37.59% (622,868) were annotated in KEGG databases. We found that the most significant KEGG pathways were Brite hierarchies (accounted for 36.52%) and metabolism (accounted for 35.05%) (Figure 4A). For level 2 KEGG orthologues, the top abundant functional pathways were protein families: genetic information processing, protein families: signaling and cellular processes, carbohydrate metabolism, protein families: metabolism, and amino acid metabolism (Figure 4B). The heatmap showed the correlation between MAGs (phylum) and KEGG pathways, which were mainly distributed in Bacillota_A, Spirochaetota, and Bacteroidota (Figure 4C). At pathway level 2, MAG374 (*Escherichia coli*, 690 pathways), MAG287 (*Novosphingobium*, 551 pathways), and MAG57 (*Arachnia* sp012837465, 447 pathways) KOs were mainly distributed in carbohydrate metabolism. The number of *Prevotella* annotated to the carbohydrate metabolism pathways was 9451, with the most prevalent being MAG756 (298 pathways) (Supplementary Table S6 and Supplementary Figure S1).

We also predicted functional capacities by calculating metabolic capacity indices (MCIs) for each MAG. In total, 17 metabolic pathways were annotated, including nucleic acid biosynthesis, amino acid biosynthesis, amino acid derivative biosynthesis, SCFA biosynthesis, organic anion biosynthesis, vitamin biosynthesis, metallophore biosynthesis, antibiotic biosynthesis, lipid degradation, sugar degradation, amino acid degradation, nitrogen compound degradation, xenobiotic degradation, antibiotic degradation, cellular structure, appendages, and spore, and 80 compounds were annotated. In detail, the top two pathways with the highest MCI were the B0103 (nucleic acid biosynthesis—UDP/UTP) and B0401 (SCFA biosynthesis—acetate). The MCI for 122 MAGs in the degradation of the sugar galactose (D0309) is 1 (Supplementary Table S7 and Supplementary Figure S2), such as MAG250 (*Ruminococcus_D sp*900314975), MAG374 (*Escherichia coli*), and MAG954 (*Limosilactobacillus equigenerosi*).

### 3.5. Analysis of Hindgut Differential MAGs

**Figure 5:**
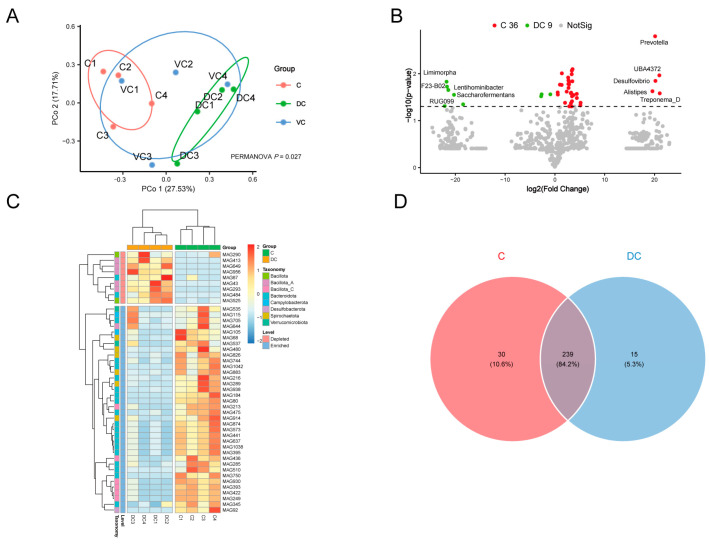
Functional profiles of the MAGs along the hindgut in Dezhou donkeys. Principal Coordinate Analysis (PCoA) based on Bray–Curtis dissimilarity of the MAGs along the hindgut in donkeys (**A**). Volcano plot (**B**). Heatmap of the differential MAGs between C and DC (**C**). Venn diagrams of unique KEGG pathways (**D**). Cecum (C), ventral colon (VC), and dorsal colon (DC).

The cecum (C), ventral colon (VC), and dorsal colon (DC) of four donkeys were selected for MAG comparative analysis. The Principal Coordinate Analysis (PCoA) revealed that the MAGs of the C and DC groups could be distinguished (*p* = 0.027) (Figure 5A). Therefore, C and DC groups were selected for further analysis, and there were 36 MAGs with significantly higher abundances in the C group using the *t*-test (*p* < 0.05) but not the Mann–Whitney test, including *Prevotella*, *UBA4372*, *Desulfovibrio*, *Alistipes*, *Treponema_D*, etc.; and 9 MAGs were higher in the DC group (*t*-test, *p* < 0.05), including *Limimorpha*, *F23-B02*, *Lentihominibacter*, *Saccharofermentans*, *RUG099*, *Lactobacillus*, etc. (Figure 5B,C).

In terms of the KEGG pathway, the C group was significantly higher in 269 pathways (36 MAGs, such as MAG1042, MAG184, MAG510, MAG80, MAG644, MAG480, MAG92, and MAG637), including 135 metabolism-related pathways, 15 carbohydrate metabolism, and 15 lipid metabolism-related pathways, including butanoate metabolism, fructose and mannose metabolism, galactose metabolism, propanoate metabolism, pyruvate metabolism, starch and sucrose metabolism, the biosynthesis of unsaturated fatty acids, fatty acid biosynthesis, and fatty acid degradation (Supplementary Table S8); the DC group was rich in 254 pathways (9 MAGs, such as MAG67, MAG290, MAG649, MAG484, MAG525, MAG413 and MAG956), including 132 metabolism-related pathways, 15 carbohydrate metabolism, and 12 lipid metabolism-related pathways (Supplementary Table S9). It is obvious that carbohydrate-related MAGs in the cecum are more plentiful and might be better at digesting plant fiber. In addition, through the richness index, Shannon index, Simpson index, and Constrained Principal Coordinate Analysis (cPCoA) based on Bray–Curtis dissimilarity, we found that the KEGG pathways were obviously segregated in the C and DC groups (*p* < 0.05) (Supplementary Figure S3).

Regarding the unique KEGG metabolic pathways, there were 30 specific metabolic pathways in the C group (Figure 5D), with functions related to the metabolic conversion and absorption of lipids, including α-linolenic acid metabolism (MAG938/MAG644), linoleic acid metabolism (MAG938/MAG644), flavone and flavonol biosynthesis (MAG80/MAG216), sesquiterpenoid and triterpenoid biosynthesis (MAG535/MAG537), steroid biosynthesis (MAG535/MAG537), fat digestion and absorption (MAG644), etc. (Supplementary Table S10). The synthesis and degradation of complex compounds, immunity, and anti-disease functions are all mediated by 15 DC-unique pathways (Figure 5D). These pathways include pathogenic Escherichia coli infection (MAG67), the degradation of fluorobenzoate (MAG67), degradation of toluene (MAG67), betalain biosynthesis (MAG484), furfural degradation (MAG956), other types of O-glycan biosynthesis (MAG649/MAG293), the degradation of polycyclic aromatic hydrocarbon (MAG649/MAG293), and Fc gamma R-mediated phagocytosis (MAG649/MAG293/MAG956) (Supplementary Table S11). It is evident that C and DC differential gut microbiota play quite different roles from each other.

### 3.6. MAGs Co-Occurrence and Interaction Networks in the Cecum

**Figure 6:**
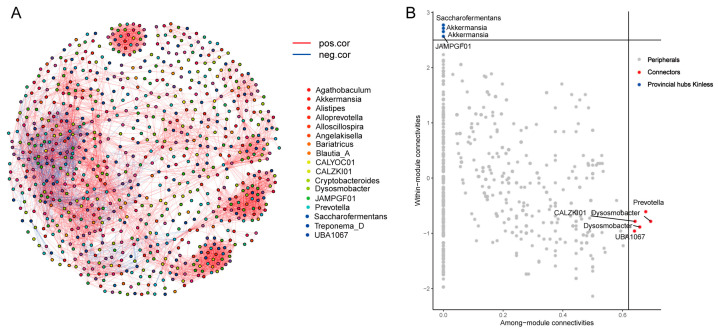
Microbial interaction networks in the cecum of donkeys. (**A**) MAG interaction networks in the cecum. (**B**) The core species were identified using the z-score and c-score.

The cecum is the primary fermentation site for donkeys; hence, it is vital to investigate the network interactions of MAGs in the cecum. As a result, we chose MAGs with an average abundance greater than 0.005% in the cecum samples, calculated Spearman correlation values between genera, z-score, and c-score values, and identified core MAGs (Supplementary Tables S12 and S13). Our findings revealed a high level of node connectivity within the cecum MAG microbiota (Figure 6A), and the further selection of core MAGs revealed that *Prevotella* and *Dysosmobacter* were highly linked among the modules (connector), while *Akkermansia* and *Saccharofermentans* nodes were highly linked within modules (provincial hubs) (Figure 6B).

## 4. Discussion

Donkeys, being monogastric herbivores, possess roughage tolerance and disease resistance, attributed to their large hindgut [18]. Previous research has emphasized the crucial role of hindgut microorganisms and their metabolites in regulating metabolism, intestinal pathogen resistance, and immune homeostasis [39]. However, there is limited research on the gut microbiota in donkeys, with most studies relying on 16S and macrogenomic sequencing methods. In recent years, the use of binning technology to reconstruct MAGs from macro-genomic samples has significantly expanded the repertoire of human and animal intestinal microbial reference genomes. This advancement has laid the foundation for further investigations into functional genes and the identification of high transformation efficiency strains [28]. Nevertheless, no reports regarding the study of donkey intestinal flora using this approach have been published to date. Therefore, our study aimed to assemble metagenomic sequences from the donkey hindgut and bin them into MAGs. This approach could substantially enhance the reference genomic information for donkey digestive tract microbes, providing a valuable resource for future research in this area.

In a previous study, we used metagenome sequencing to characterize the microbial community and its functional potential in the cecum and colon of donkeys [18]. In this report, we present the assembly and analysis of 844 MAGs obtained from these data. Among these MAGs, 678 represent species that are not closely related to any genomes in the reference database (ANI < 95%), indicating that they may be novel species. These MAGs greatly enhance the collection of microbial reference genomes available for this ecosystem. The predominant phyla observed in the donkey gut microbiota in our study were Bacillota (403 MAGs) and Bacteroidota (309 MAGs). These findings are consistent with recent studies that used 16S rRNA gene sequencing and metagenome analysis [18]. Additionally, we found that *Cryptobacteroides* and *Prevotella* were the most abundant genera, which aligns with previous research [40].

In our study, we found only 3 MAGs of archaea among the 844 MAGs obtained, which may be due to the influence of data analysis factors such as small sample size or low sequencing depth, in addition to host factors, external environment and the characteristics of the archaea themselves may affect the results resulting in a lower number of archaea. The gut microbiomes of different species are highly specific [41,42], and the evolutionary history and habits of the donkey, as a unique monogastric animal, may have led to the formation of ecological niches in the gut that favor specific bacterial communities [7]. The composition of the feed directly affects the type and number of gut microbes. Donkeys consume mainly plant-based fibers, so bacteria that break down cellulose will be more active [15], while certain archaea that depend on specific substrates may be relatively scarce. Depending on the characteristics of the archaea themselves, the bacteria may have a competitive advantage in a complex microbial community, thus inhibiting the growth of the archaea.

The hindgut of the donkey shares similarities with the rumen of ruminants in both structure and function. These similarities enable the donkey to break down plant polysaccharides. The abundance of microbial populations in the hindgut is closely linked to this process. The gut microbiota of donkeys contains a significant number of enzymes called CAZymes that are responsible for the degradation and metabolism of polysaccharides [43]. Notably, a total of 292,980 CAZymes, representing 17.68% of the total predicted proteins, were annotated in this study, which is significantly higher than the proportion predicted for horse intestinal MAGs (6.77%) [22]. This observation suggests that the donkey’s heightened tolerance to roughage may be attributed to its increased CAZyme abundance [44]. Verrucomicrobiota identified MAGs (MAG49, MAG623, and MAG933, belonging to undescribed strains, ANI < 95%) that encode the most CAZymes and can degrade various glycans, such as cellulase (GH5 and GH9), fucosidase (GH29), and pectate lyase (PL1). Consistent with previous findings, Orellana et al. also found that MAGs annotated to Verrucomicrobiota had the highest number of GHs, as well as having carried the highest content of fucosidases and rhamnosidases [45]. Lachnospiraceae KLE1615 Unassigned (MAG774, ANI < 95%) is annotated to 725 PULs and numerous CAZymes, including β-fructofuranosidase (GH43), cellulase (GH5 and GH9), pectate lyase (PL3), β-glucosidase (GH3), β-1,4-xylosidase (GH51), α-1,3-L-galactosidase (GH29), and β-1,3-xylanase (GH8). Additionally, MAGs (such as *Prevotella* Unassigned (MAG207, MAG19, and MAG376), UBA4334 Unassigned (MAG898), *Cryptobacteroides* Unassigned (MAG175, MAG506, and MAG79) and *Phocaeicola faecalis* (MAG728)) from Bacteroidales were annotated to a surprisingly high number of CAZymes. Bacteria in this repertoire contain numerous and plentiful CAZymes, which can be called polysaccharide utilization sites (PULs). It is commonly known that bacteria in this class have an enormous and varied repertoire of CAZymes, which can be arranged into clusters of genes known as PULs [46], of which *Prevotella* (e.g., MAG19, MAG 207, MAG 376, MAG 737, and MAG 756) annotated both a significant number of PULs (more than >500) and abundant CAZymes, such as α-amylases (GH13 and GH31) and mannosidase (GH38), involved in the breakdown of starch and oligosaccharides. α-arabinofuranosidase (GH51), α-fucosidase (GH29, GH18), β-xylanase (GH43), xylan esterase (GH26), 1,4-α-glucan branching enzyme (CBM48), and cellulase (GH5, GH9, GH26) were found to hydrolyze hemicellulose and cellulose, also annotated to PULs that degrade the aforementioned polysaccharides, as were PUL0239 and PUL0240 (degradation cellulose), and PUL0342 and PUL0456 (degradation xylan). In addition, 298 pathways (MAGs 756) were annotated at KEGG level 2 carbohydrate metabolism pathways, including α-1,2-mannosidase, α-galactosidase, α-L-fucosidase, β-galactosidase, β-xylanase, cellulase, glucuronate isomerase, glycosyl hydrolase, mannitol dehydrogenase, pectate lyase, pectinesterase, and polysaccharide deacetylase, which could be connected to the way *Prevotella* breaks down complex polysaccharides such as cellulose and hemicellulose [47]. This suggests that *Prevotella* may play essential roles in lignocellulose degradation in the hindgut of donkey. Previous studies have demonstrated that *Prevotella* possesses a considerable amount of CAZymes and numerous PULs [48,49,50], as well as the ability to degrade complex polysaccharides such as hemicellulose and cellulose [51]. In accordance with previous studies, *Phocaeicola* faecalis possesses certain CAZymes including GH20, GH29, PL8, and PL8_3, which are capable of breaking down polysaccharides to produce SCFA (acetate and butyrate), thereby decreasing intestinal inflammation in mice [52]. These findings are consistent with the current study. This study annotated undescribed (ANI < 95 or NA) MAGs from *Kiritimatiellia*, *Lachnospiraceae*, *Prevotella*, *Cryptobacteroides*, and *Phocaeicola* that have not yet been cultured. The complete genome data provided in this study are crucial for further research into the degradation of complex polysaccharides in donkey hindgut. Furthermore, both CAZymes (99.70%) and PULs (99.94%) identified in this study matched less than 50% homology with current databases, indicating that the CAZymes and PULs of donkey hindgut microbes are absent from public databases. This suggests that there is still much to learn and discover about novel carbohydrate-active enzymes and microbial species in the donkey’s hindgut.

According to a recent study on metagenomic sequences [18], the PCoAs showed significant differentiation among the reconstructed MAGs in the donkey cecum (C) and dorsal colon (DC), suggesting a clear difference in the microbial composition between these two gastrointestinal regions. Additionally, the functional analysis revealed distinct metabolic pathways that were distributed in the cecum and DC. Specifically, the cecum showed the main pathways associated with lipid metabolism and absorption, while the DC exhibited the primary pathways related to the synthesis and degradation of complex compounds, as well as immune response and disease prevention mechanisms [53]. These differences likely correspond to the different physiological roles of the cecum and DC. However, further research is needed to understand the potential implications of these pathways on the host’s health.

Based on metabolic functions, bacteria in the cecum can be classified as cellulolytic, proteolytic, lactate-using, and glycolytic [11]. Among these, *Prevotella* is a genus that is significantly more abundant in the cecum. It includes strains such as MAG1042, MAG184, MAG510, and MAG80. *Prevotella* is primarily involved in the metabolism of volatile fatty acids, including butanoate, propanoate, and pyruvate. It also plays a crucial role in carbohydrate metabolism including fructose, mannose, galactose, starch, and sucrose metabolism. *Prevotella* is also active in lipid metabolism, participating in processes such as the biosynthesis of unsaturated fatty acids, fatty acid biosynthesis, fatty acid degradation, glycerolipid metabolism, glycerophospholipid metabolism, and the PPAR signaling pathway. Previous studies have highlighted the involvement of *Prevotella* in lipid metabolism and the PPAR signaling pathway in the donkey cecum, suggesting its potential role in regulating lipid metabolism and influencing the host’s fat deposition [18,54]. Additionally, *Prevotella* is related in the metabolic pathways of oligosaccharides and starch, attributable to its extensive repertoire of CAZymes [55]. This study also identifies *Prevotella* in the metabolic pathways of butyrate and propionate. Such findings underscore the importance of *Prevotella* in the production and utilization of volatile fatty acids, particularly butyrate and propionate, thus contributing to the gut homeostasis in donkeys [56]. In contrast, the genus *Desulfovibrio* (MAG92, MAG461, and MAG837, ANI < 95%) is prevalent in the cecum and known for its ability to reduce sulfate to hydrogen sulfide (H_2_S), a process associated with intestinal inflammation [57]. The KEGG pathway analysis in this study also revealed the involvement of *Desulfovibrio* in sulfur metabolism (ko00920), highlighting its potential role in the donkey cecum, which requires further investigation. *Alistipes*, which is associated with intestinal dysbiosis and disease [58], has been shown to promote pectin degradation [59]. In this study, the reconstructed *Alistipes* MAG938 (ANI < 95%) was found to be annotated to pathways involved in cellulase and β-galactosidase, which may be related to dietary fiber degradation in the donkey cecum.

*Treponema*, a member of the Spirochaetes phylum, is more abundant in the cecum. Treponema is closely associated with cellulose digestion and utilization [60] as well as hemicellulose degradation [61]. The reconstructed Treponema MAGs (MAG100, MAG108, MAG1005, and MAG1019, ANI < 95%) in this study were annotated to several pathways associated with carbohydrate degradation, including cellulase, β-glucosidase, α-1,4-glucan branching enzyme, and α-amylase. *Saccharofermentans* (MAG413), which is more abundant in the dorsal colon, has been linked to polysaccharide breakdown and utilization. Previous studies indicate that *Saccharofermentans* can catabolize polysaccharides to produce VFAs [62], which may contribute to fiber utilization in the dorsal colon. Furthermore, Lactobacillus (*Lactobacillus amylovorus*, MAG1027; *Lactobacillus crispatus*, MAG237; *Lactobacillus equicursoris*, MAG290; MAG525, unidentified species, ANI < 95%) was found in greater abundance in the dorsal colon compared to the cecum. Pathways such as glyoxylate and dicarboxylate metabolism (ko00630), propanoate metabolism (ko00640), and butanoate metabolism (ko00650) were identified, potentially associated with the higher concentrations of VFAs in the dorsal colon [18].

The cecum makes up about 12–15% of the total capacity of the gastrointestinal tract. It contains a dense population of anaerobic bacteria, with concentrations ranging from 1.85 × 10^7^ to 2.65 × 10^9^ cfu/mL [11]. These bacteria form complex ecological networks through interactions such as cooperation, competition, and predation [63]. Our study reveals a strong connectivity among the bacterial species in the cecum, with *Prevotella* and *Dysosmobacter* identified as key connectors with high intermodular connectivity. *Akkermansia* and *Saccharofermentans*, on the other hand, show significant intramodular connections and function as provincial hubs. *Prevotella*, in particular, plays a central role in cooperative and competitive interactions with various components of the gut microbiota, making it a keystone taxon [64]. Meanwhile, *Dysosmobacter*, a relatively understudied bacterium known for its production of butyric acid, has shown promise in mitigating diet-induced obesity and metabolic dysfunctions in murine models, suggesting its potential as a novel probiotic [65]. The presence of *Prevotella* and *Dysosmobacter* likely has a significant impact on the structure and function of the donkey gut microbiota. *Akkermansia*, on the other hand, has been associated with a lower the risk of inflammatory bowel disease, obesity, and type 2 diabetes mellitus [66]. Our study also confirms *Akkermansia* as a provincial hub strain in the donkey cecum, consistent with previous research showing its unique presence in healthy individuals and its role in host immune function [67,68]. *Saccharofermentans*, with its ability to degrade polysaccharides and produce volatile fatty acids, plays a crucial role in fiber utilization and intestinal health in the hindgut of the donkey. This bacterium is important for regulating the structure and function of the microbial community in the donkey’s gastrointestinal tract [69]. However, further research is needed to understand the mechanisms and roles of these connector and hub species in donkeys. Our study provides a comprehensive genome-wide assembly of these bacteria, establishing a foundation for future investigations into their functional roles in the donkey gastrointestinal tract.

## 5. Conclusions

This study provides a comprehensive analysis of the donkey hindgut microbiome through metagenomic sequencing of donkey gut samples. A total of 844 non-redundant MAGs were identified, revealing a rich and previously underexplored microbial diversity. Notably, 80% of these MAGs correspond to novel, undescribed species. The functional analysis highlighted a remarkable array of CAZymes and PULs, many of which are unique, indicating the donkey’s ability to digest complex plant fibers. Additionally, differential microbial abundances in the cecum and dorsal colon were observed, with distinct microbial communities associated with different metabolic pathways, particularly those involved in carbohydrate and lipid metabolism. Network analysis revealed key genera, such as *Prevotella* and *Dysosmobacter*, as crucial players in complex carbohydrate degradation. These findings not only deepen our understanding of the donkey gut microbiome but also highlight novel microbial functions, offering valuable insights into hindgut metabolism and potential applications in animal nutrition and health.

## Data Availability

The unassembled sequences as well as the MAGs from the present study were submitted to the NCBI Sequence Read Archive under accession number PRJNA860652 and PRJNA1103961.

## Supplementary Materials

The following supporting information can be downloaded at https://www.mdpi.com/article/10.3390/ani14243625/s1: Figure S1: Number of KEGG pathway annotations in level 2 (carbohydrate metabolism) of MAGs; Figure S2: Heatmap of predicted functional capacities by metabolic capacity indices (MCIs) for each MAG; Figure S3: α and β diversity of KEGG pathways in the C, VC, and DC groups. Table S1: All detailed information on experimental donkeys and samples; Table S2: Taxonomy and assembly statistics of metagenome-assembled genomes (MAGs) in the hindgut microbiome of donkeys; Table S3: Number of carbohydrate-active enzymes (CAZymes) and CAZyme families for each metagenome-assembled genome (MAG); Table S4: Eggnog CAZyme annotation of each MAG; Table S5: Number of polysaccharide utilization loci (PULs) for each metagenome-assembled genome (MAG); Table S6: Number of KEGGs in level 2 for each metagenome-assembled genome (MAG); Table S7: Metabolic capacity indices (MCIs) for each metagenome-assembled genome (MAG); Table S8: Number of KEGG pathways significantly higher in cecum; Table S9: Number of KEGG pathways significantly higher in dorsal colon; Table S10: Number of KEGG pathways significantly higher in cecum (unique pathways); Table S11: Number of KEGG pathways significantly higher in dorsal colon (unique pathways); Table S12: Correlation between the differential MAGs in cecum; Table S13: z-score and c-score values of MAGs in cecum.
