# Supplementary material for: Insights into the Donkey Hindgut Microbiome Using Metagenome-Assembled Genomes
Source: Animals (Basel). 2024 Dec 16;14(24):3625. doi: 10.3390/ani14243625 (PMC11672655; doi:10.3390/ani14243625)
Supplement: Supplementary file 1 [file animals-14-03625-s001.zip › Data Sheet 1.pdf]

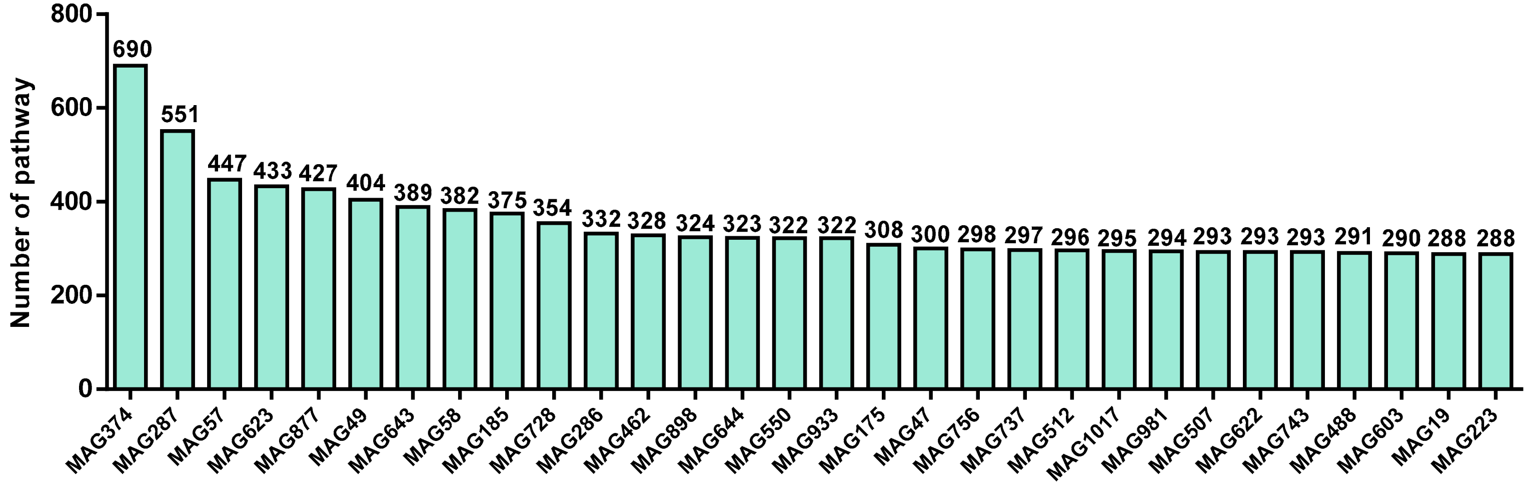

Supplementary Figure S1. Number of KEGG pathways annotation in level 2 (carbohydrate metabolism) of MAGs.

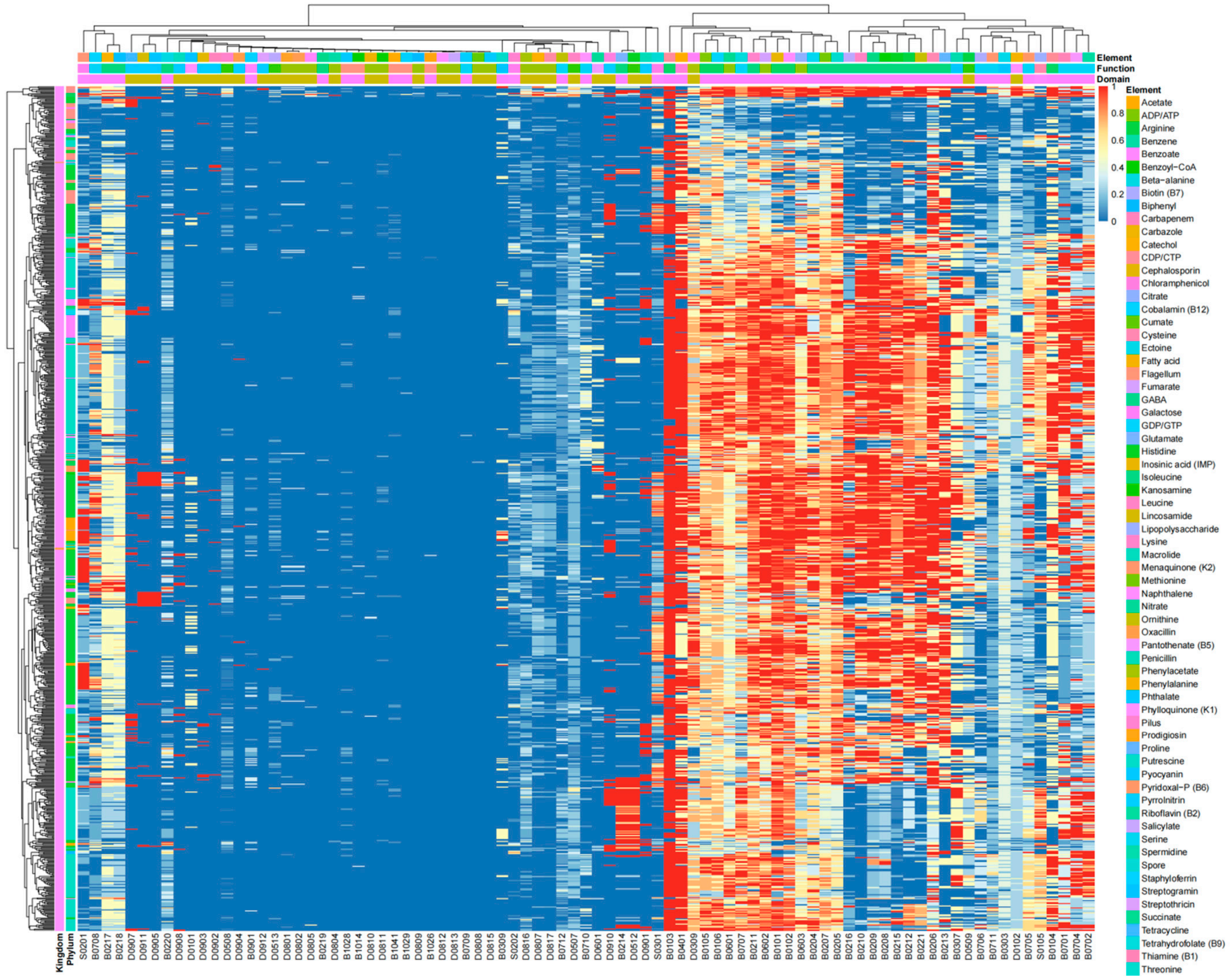

Supplementary Figure S2. Heatmap of predicted functional capacities by Metabolic Capacity Indices (MCIs) for each MAG. Values closer to 1 (dark red) indicate a higher capacity, while values closer to 0 (dark blue) indicate lower or no capacity.

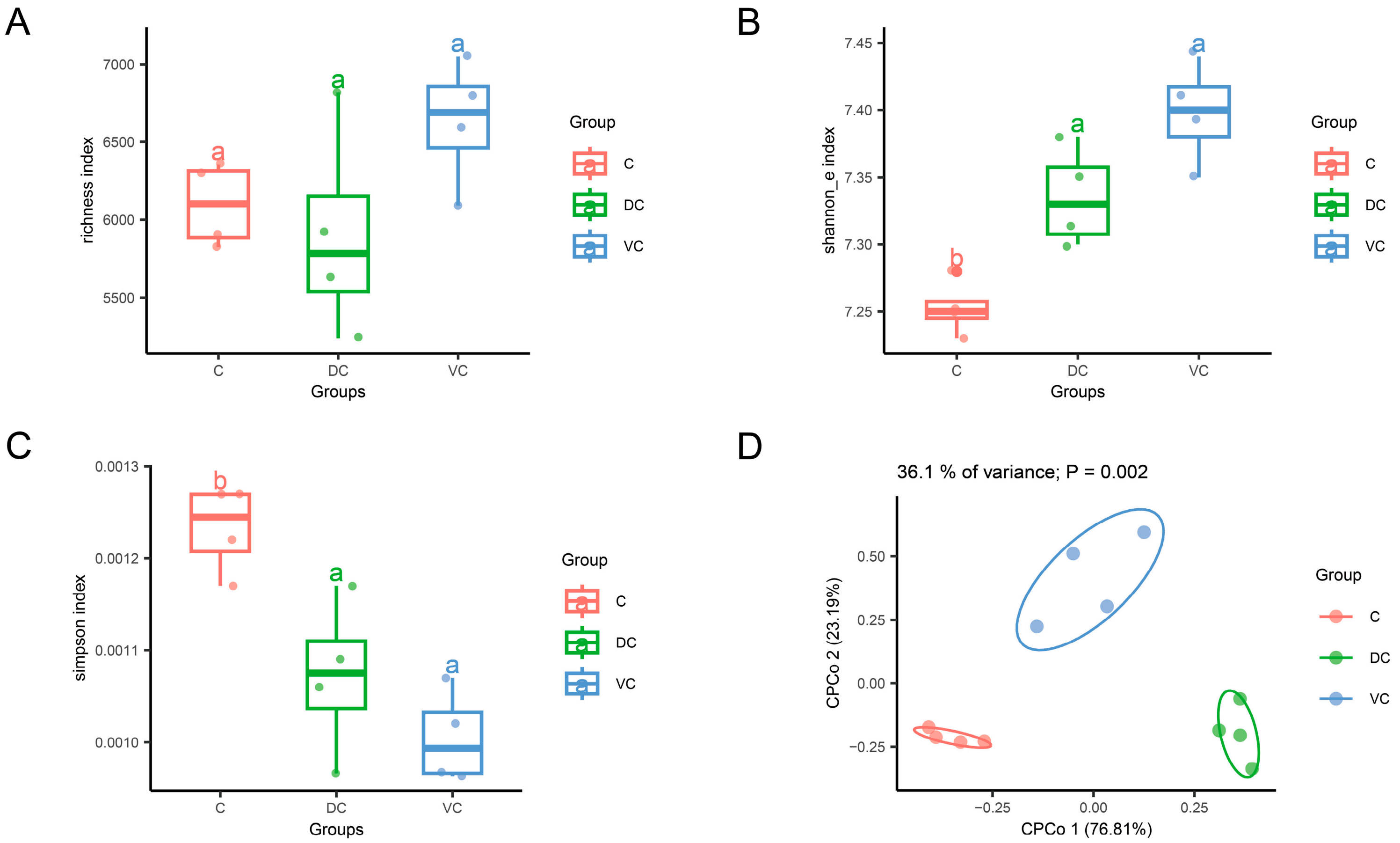

Supplementary Figure S3. α and β diversity of KEGG pathways in the C, VC and DC groups. A, richness index; B, shannon index; C, simpson index; and D, Constrained principal coordinates analysis (cPCoA) and PERMANOVA test (P = 0.002). C, cecum; VC, ventral colon; DC, dorsal colon
